# Predicting Outcomes of Language Rehabilitation: Prognostic Factors for Immediate and Long-Term Outcomes After Aphasia Therapy

**DOI:** 10.1044/2022_JSLHR-22-00347

**Published:** 2023-02-24

**Authors:** Sigfus Kristinsson, Alexandra Basilakos, Dirk B. den Ouden, Christy Cassarly, Leigh Ann Spell, Leonardo Bonilha, Chris Rorden, Argye E. Hillis, Gregory Hickok, Lisa Johnson, Natalie Busby, Grant M. Walker, Alexander McLain, Julius Fridriksson

**Affiliations:** a Center for the Study of Aphasia Recovery, University of South Carolina, Columbia; b Department of Public Health Sciences, Medical University of South Carolina, Charleston; c Department of Neurology, Medical University of South Carolina, Charleston; d Department of Psychology, University of South Carolina, Columbia; e Department of Physical Medicine and Rehabilitation, Johns Hopkins School of Medicine, Johns Hopkins University, Baltimore, MD; f Department of Cognitive Science, Johns Hopkins University, Baltimore, MD; g Department of Cognitive Sciences, School of Social Sciences, University of California, Irvine; h Department of Epidemiology and Biostatistics, University of South Carolina, Columbia

## Abstract

**Background:**

Aphasia therapy is an effective approach to improve language function in chronic aphasia. However, it remains unclear what prognostic factors facilitate therapy response at the individual level. Here, we utilized data from the POLAR (Predicting Outcomes of Language Rehabilitation in Aphasia) trial to (a) determine therapy-induced change in confrontation naming and long-term maintenance of naming gains and (b) examine the extent to which aphasia severity, age, education, time postonset, and cognitive reserve predict naming gains at 1 week, 1 month, and 6 months posttherapy.

**Method:**

A total of 107 participants with chronic (≥ 12 months poststroke) aphasia underwent extensive case history, cognitive–linguistic testing, and a neuroimaging workup prior to receiving 6 weeks of impairment-based language therapy. Therapy-induced change in naming performance (measured as raw change on the 175-item Philadelphia Naming Test [PNT]) was assessed 1 week after therapy and at follow-up time points 1 month and 6 months after therapy completion. Change in naming performance over time was evaluated using paired *t* tests, and linear mixed-effects models were constructed to examine the association between prognostic factors and therapy outcomes.

**Results:**

Naming performance was improved by 5.9 PNT items (Cohen's *d* = 0.56, *p* < .001) 1 week after therapy and by 6.4 (*d* = 0.66, *p* < .001) and 7.5 (*d* = 0.65, *p* < .001) PNT items at 1 month and 6 months after therapy completion, respectively. Aphasia severity emerged as the strongest predictor of naming improvement recovery across time points; *mild* (ß = 5.85–9.02) and *moderate* (ß = 9.65–11.54) impairment predicted better recovery than *severe* (ß = 1.31–3.37) and *very severe* (ß = 0.20–0.32) aphasia. Age was an emergent prognostic factor for recovery 1 month (ß = −0.14) and 6 months (ß = −0.20) after therapy, and time postonset (ß = −0.05) was associated with retention of naming gains at 6 months posttherapy.

**Conclusions:**

These results suggest that therapy-induced naming improvement is predictable based on several easily measurable prognostic factors. Broadly speaking, these results suggest that prognostication procedures in aphasia therapy can be improved and indicate that personalization of therapy is a realistic goal in the near future.

**Supplemental Material:**

https://doi.org/10.23641/asha.22141829

Stroke is the most common cause of long-term disability in Western societies (Adamson et al., 2004). It affects up to 800,000 individuals in the United States alone each year (Virani et al., 2020) and commonly results in aphasia, a devastating language disorder. Many individuals regain lost language function in the months following stroke onset (Lazar et al., 2010; Pedersen et al., 1995), but for approximately a third of stroke survivors, aphasia becomes a chronic condition (Flowers et al., 2016). Aside from the obvious implications of aphasia on all communicative aspects of daily life, aphasia negatively affects health outcomes (Lazar & Boehme, 2017), employment (Graham et al., 2011), and mental health (Kauhanen et al., 2000) and is associated with worse quality of life than any other neurologic disorder (Lam & Wodchis, 2010). The rising incidence rate of stroke in younger populations, accompanied by increased life expectancy (Joundi et al., 2021; Li et al., 2020; Wafa et al., 2020), has precipitated higher prevalence of chronic aphasia than ever before (Simmons-Mackie, 2018). Consequently, the demand for evidence-based resources to improve poststroke language outcomes has increased markedly in recent years.

In this study, we present data from the POLAR (Predicting Outcomes of Language Rehabilitation in Aphasia) trial (clinicaltrials.gov identifier: NCT03416738). POLAR is a large-scale clinical trial focused on predictors of therapy-induced language outcomes in chronic aphasia by incorporating a relatively larger study sample than most previous therapy trials and through rigorous testing and therapy protocols. Briefly, the purpose of the POLAR trial is to identify prognostic factors for therapy outcomes and leverage this knowledge to maximize the efficacy of personalized aphasia treatment. To this end, two types of treatments were applied in a randomized crossover design, one phonologically focused and the other semantically focused. In order to guarantee identical therapy administration for all participants, irrespective of aphasia severity, both treatments aimed to strengthen lexical–semantic access by focusing specifically on evidence-based naming tasks. This therapy focus was motivated by the fact that all individuals with aphasia experience impaired lexical–semantic access, that is, anomia, to some degree (Goodglass & Wingfield, 1997). As such, progress was similarly assessed as treatment-induced change in naming performance. Consistent with the main aim of POLAR, we reported factors associated with response to phonological and semantic therapies in a recent publication (Kristinsson, Basilakos, et al., 2021). Building on this work, this study focuses explicitly on prognostic factors that have been associated with the general capacity to recover lost language function through rehabilitation, irrespective of therapy type.

Behavioral speech and language therapy (SLT) has eloquently been shown to be an effective approach to restoring language function in chronic aphasia (≥ 6 months poststroke; Brady et al., 2012, 2016; Breitenstein et al., 2017; Fridriksson & Hillis, 2021). As such, SLT remains the standard of care in the clinical management of aphasia (Fama & Turkeltaub, 2014). However, despite positive therapeutic effects reported at the group level, there is notorious and largely unexplained variability in language recovery at the level of the individual (e.g., Code et al., 2010; Fridriksson et al., 2012; Palmer et al., 2012; Szaflarski et al., 2015). For this reason, personalized therapy planning with a certain degree of confidence about the expected outcome has remained elusive in aphasia therapy.

Considerable research effort has been undertaken to investigate personal factors associated with favorable response to SLT (e.g., Fridriksson et al., 2012; L. Johnson et al., 2019; Lambon Ralph et al., 2010; Meier et al., 2019). These studies have consistently implicated initial aphasia severity as an important prognostic factor (Basso, 1992; Fridriksson & Hillis, 2021; Nakagawa et al., 2019). Other frequently investigated factors include age (e.g., Ali et al., 2015; Hillis et al., 2018), education (e.g., Hillis et al., 2018; Hillis & Tippett, 2014), time postonset (TPO; e.g., Basso, 1992; Moss & Nicholas, 2006), and cognitive reserve (e.g., Dignam et al., 2017; Lambon Ralph et al., 2010). However, these efforts have not resulted in generalizable predictors of therapy outcomes to date (e.g., Fridriksson & Hillis, 2021; Plowman et al., 2012; Watila & Balarabe, 2015). One of the primary reasons for the evident scarcity of concrete findings in this area relates to the fact that most previous studies have incorporated too few study participants to substantiate the statistical power necessary to make reliable inferences in such a heterogeneous population (Lorca-Puls et al., 2018). This was strikingly demonstrated in a recent literature review on treatment-related brain changes in aphasia; only 2/32 studies included over 20 participants (Schevenels et al., 2020). Thus, even if there is consensus within the field that SLT is effective at the group level, it remains largely unknown how to maximize the effectiveness of therapy for any given individual.

As a result, prognostication is challenging in the clinical management of chronic aphasia (Cheng et al., 2020; Tierney-Hendricks et al., 2021). This reality, in conjunction with the rising prevalence of chronic aphasia, has introduced a rapidly growing demand for a more personalized approach to aphasia therapy in recent years (e.g., Berube & Hillis, 2019; Doogan et al., 2018; Kiran & Thompson, 2019). The development of personalized aphasia therapy requires a detailed understanding of how various personal characteristics impact the neuroplastic processes that support the therapy-induced reorganization of language.

This study echoes this perspective. Specifically, in addition to describing in detail the methodology of the POLAR trial, we aimed to (a) determine therapy-induced change in confrontation naming and long-term maintenance of naming gains and (b) examine the extent to which aphasia severity, age, education, TPO, and cognitive reserve predict language gains (measured as change in confrontation naming) 1 week, 1 month, and 6 months posttherapy. The implications of the current findings are subsequently discussed in the context of the extant aphasia therapy literature.

## Method

### Participants

Participants were eligible for recruitment if they were between 21 and 80 years of age and had chronic aphasia (≥ 12 months postonset) due to left-hemisphere ischemic or hemorrhagic stroke. Individuals with multiple strokes were eligible for study participation as long as all structural lesions were confined to the left supratentorial hemisphere. Participants were excluded if they had severely limited speech output (Western Aphasia Battery–Revised [WAB-R]; Kertesz, 2007; WAB-R Spontaneous Speech rating scale score of 0–1), severely limited auditory comprehension (WAB-R Auditory Comprehension score of 0–1), contraindications for magnetic resonance imaging, and/or a bilateral stroke. Study procedures were carried out at the University of South Carolina and the Medical University of South Carolina, and this study was approved by the institutional review boards at both universities. All participants consented to all study procedures.

### Procedure

Study activities took place over a 9-month period from the time of baseline assessments through the final assessment time point 6 months after therapy completion. Prior to therapy, participants underwent a medical history interview, neuroimaging workup, and a comprehensive cognitive–linguistic baseline assessment, including the following instruments: Apraxia of Speech Rating Scale (Strand et al., 2014) to determine the presence and severity of apraxia of speech, National Institutes of Health Stroke Scale (Brott et al., 1989) to assess stroke severity, Philadelphia Naming Test (PNT; Roach et al., 1996) to measure naming performance, Philadelphia Repetition Test (Dell et al., 1997; Roach et al., 1996) to evaluate speech repetition and phonological processing, Pyramids and Palm Trees Test (Howard & Patterson, 1992) to assess nonverbal semantic processing, Wechsler Adult Intelligence Scale (WAIS) Matrix Reasoning subtest (Wechsler, 2008) for nonverbal reasoning, and WAB-R to measure aphasia severity. A detailed breakdown of baseline test scores for all participants is presented in Supplemental Material S1 (see Kristinsson, Basilakos, et al., 2021, for a complete overview of cognitive–linguistic assessments in the POLAR repository).

**Figure 1.**
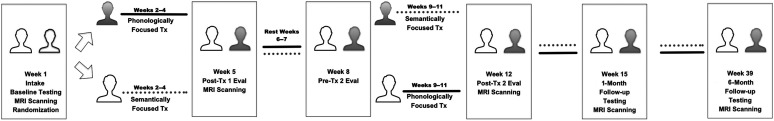
Study timeline. MRI = magnetic resonance imaging; Tx = treatment.

Following baseline assessments, each participant was assigned to one of two treatment groups following the asymptotic maximal procedure in a 1:1 ratio (Zhao et al., 2018): phonological therapy followed by semantic therapy or semantic therapy followed by phonological therapy. Each therapy phase lasted 3 weeks, and therapy was delivered 5 days per week for an hour per day. Thus, all participants received a total of 15 hr of phonological therapy and 15 hr of semantic therapy. After the first therapy phase, participants underwent outcome assessments and a second neuroimaging workup, followed by 2 weeks of rest from all study activities. Participants returned for a behavioral assessment preceding the second therapy phase. In the week following completion of therapy, participants underwent an outcome assessment and a third neuroimaging workup, as well as identical evaluations 1 and 6 months after therapy. Participants were restricted from participating in other therapeutic activities from entry to this study through the 6-month follow-up. Figure 1 illustrates the study timeline.

### Aphasia Therapy

Participants received a total of 30 hr of therapy, dispersed over 6 weeks. All therapy tasks employed in the POLAR trial have been subjected to empirical testing in prior research. Two sets of 60 words were trained, one for phonological and one for semantic therapy. Both lists included 50 nouns and 10 verbs ranging from one to four syllables. The word lists were matched for linguistic complexity and word frequency. American Speech-Language-Hearing Association (ASHA)–certified speech-language pathologists (SLPs) with experience working with individuals with aphasia delivered all therapy tasks. Assessment and therapy fidelity was monitored by another ASHA-certified SLP to ensure consistency in therapy delivery among all SLPs (author L.A.S.; additional details provided in Spell et al., 2020).

The phonologically focused therapy consisted of three therapy tasks: phonological components analysis (PCA; Leonard et al., 2008), a phonological production task (Cubelli et al., 1988; J. Marshall et al., 1998), and a custom-designed computerized phonological judgment task. In the PCA task, the participant was asked to name a picture and to identify phonological features of the target word (e.g., first sound, syllables, last sound, association, and rhyme). Prior to moving on to the next item, the participant was prompted to attempt to name the picture again. The phonological production task focused on the identification of phonological features using a stack of targeted imageable nouns and verbs. As a first step, the participant was asked to sort the pictures based on the number of syllables by tapping out each syllable. After the participant had sorted the stimulus words into two stacks, they were asked to identify the following hierarchy of phonological features for noun–verb target pairs: (a) first syllable–first syllable, (b) first syllable–last syllable, (c) last syllable–last syllable, (d) last syllable–first syllable, (e) first syllable–first sound, (f) last syllable–last sound, (g) first syllable–last sound, and (h) last syllable-first sound. Once each targeted feature was identified for the pair of words, the participant was required to blend the syllables/sounds together. Finally, the phonological judgment task relied on computerized presentation of verbs and nouns where participants were required to judge whether pairs of words included similar phonological features. The task comprised five conditions that entailed determining (a) if a set of words included the same number of syllables, (b) if a set of words included the same initial syllable, (c) if a set of words included the same final syllable, (d) which word had more syllables, and (e) rhyme. Participants responded to each condition by pressing one of two response buttons depending on the task requirements and instructions.

The semantically focused therapy similarly consisted of three tasks: semantic feature analysis (SFA; Boyle, 2004; Boyle & Coelho, 1995), a modified version of the Promoting Aphasics' Communication Effectiveness (Davis, 2005) semantic barrier task, and Verb Network Strengthening Treatment (VNeST; Edmonds et al., 2009). In the SFA task, the participant was prompted to produce words semantically related to a presented target word (e.g., superordinate category, use, action, physical properties, location, and association). For example, to elicit a location feature, the clinician might say, “Where do you typically find this object?” If the participant was not able to name the target item once each word feature had been produced, the clinician produced the target word. Regardless of naming accuracy on the last item, treatment continued on to the next target word. Both nouns and verbs were used in the task. The semantic barrier task relied on a stack of picturable stimuli, which were split between the participant and the clinician and placed face up on a table. A visual barrier was placed between the clinician and the participant, so they were unable to see each other's pictures. The goal of the task was for one player (e.g., participant) to describe each card so that the other player (e.g., clinician) could guess the picture on the card, and vice versa. Players were only allowed to describe the semantic features of the target, and the clinician modeled the kinds of cues that were allowed. The third approach, VNeST, is a semantic treatment approach that targets lexical retrieval of verbs and their thematic nouns. The objective of VNeST was for the participant to generate verb–noun associates with the purpose of strengthening the connections between the verb and its thematic roles. VNeST can be modified to fit participants with very limited speech output (e.g., using sentence completion). Readers are referred to the aforementioned citations for additional details regarding each therapy approach.

It is worth noting that although each therapy task has been shown to be effective on its own, the tasks have not been studied together to the authors' knowledge (e.g., SFA and VNeST). There are two main reasons for the choice to apply the tasks together as opposed to using a single task. First, we aimed to maximize semantic and phonological processing demands by applying a multifaceted therapy approach. Second, these tasks are rarely implemented in isolation across consecutive therapy sessions. Since the POLAR trial was designed to replicate clinical practice as well as reasonably possible, different tasks with the same focus were combined to approximate routine outpatient therapy procedures.

### Outcome Assessment

Performance on the PNT, a measure of object picture naming, served as the primary outcome for evaluating change in untrained naming. The PNT was scored according to guidelines provided by Roach et al. (1996). To account for day-to-day variability in aphasic performance, the PNT was administered on two consecutive days at baseline, and performance was averaged across days (test–retest ρ = .99, *p* < .001). A single PNT assessment was carried out 1 week, 1 month, and 6 months posttherapy. Graduate research assistants, blind to participant details and order of therapy phase, transcribed and coded all samples. These students were closely supervised by an ASHA-certified SLP (author L.A.S.) and researchers with extensive experience rating language samples obtained from individuals with aphasia. Excellent inter- and intrarater reliability was maintained throughout the study period (Spell et al., 2020).

### Data Availability

**Figure 2.**
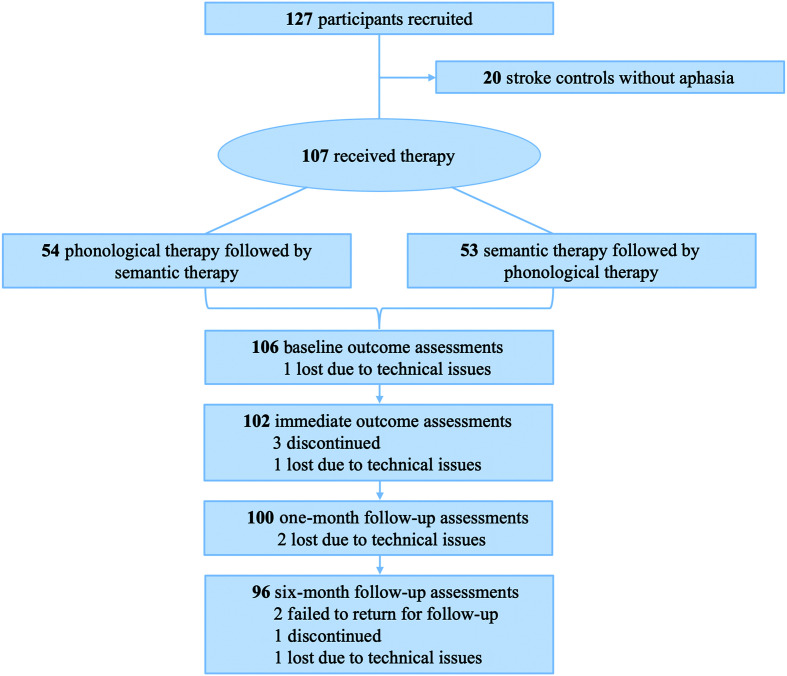
Participant flow diagram.

One participant was missing a baseline PNT score due to a video error that affected off-line scoring. Three individuals discontinued study participation prior to the posttherapy outcome assessments, and the 1 week posttherapy PNT score for one participant was missing due to technical issues. At the 1-month follow-up assessment, two PNT assessments were missing due to a video error. At the 6-month follow-up, one additional participant was discontinued in this study, one video recording was lost due to technical issues, and two participants failed to return for the follow-up assessment. Figure 2 presents a flowchart of data availability at each assessment time point.

A total of 102 participants who completed baseline assessments and at least one outcome assessment were included in data analyses. The data that support the findings of this study are available from the corresponding author upon reasonable request.

### Statistical Analysis

Although the POLAR trial was not designed to investigate therapy efficacy as such, we first assessed therapy-induced change in naming to confirm the expected response to established SLTs. To this end, naming performance on the PNT 1 week, 1 month, and 6 months posttherapy was compared to baseline performance. Paired *t* tests were used to compare PNT scores at each outcome assessment with baseline performance. Similarly, PNT scores were compared across all outcome assessment time points. In total, we performed six pairwise *t* tests (baseline vs. 1 week, 1 month, 6 months; 1 week vs. 1 month, 6 months; 1 month vs. 6 months). A Bonferroni-corrected statistical threshold was therefore set at *p* < .008.

Second, we investigated the relationship between five prognostic factors and treated language recovery: Western Aphasia Battery–Aphasia Quotient (WAB-AQ) as a measure of aphasia severity, age, TPO in months, education in years, and WAIS Matrix Reasoning score as a measure of cognitive reserve. Relationships across independent variables (i.e., WAB-AQ, age, TPO, education, and WAIS Matrix Reasoning) and between independent variables and naming performance at baseline, as well as long-term change in naming performance (1 week, 1 month, and 6 months posttherapy), were inspected using Pearson's or Spearman's correlation as appropriate. This analysis served to characterize the relationship across variables. For this reason, we report the results without correction for multiple comparisons.

Third, we constructed general linear models (GLMs) to test the association between predictor variables and therapy-induced naming gains. Consistent with recent findings indicating that prognostic factors for immediate therapy response may differ from those of long-term retention of therapeutic gains (Breitenstein et al., 2017; Pompon et al., 2017), we constructed separate GLMs for the change in PNT score from baseline to 1 week, 1 month, and 6 months posttherapy. Four of the five prognostic factors were treated as continuous variables, but aphasia severity was treated as a categorical variable with four levels (WAB-AQ = 0–25, *very severe*; 26–50, *severe*; 51–75, *moderate*; ≥ 76, *mild*; Kertesz, 2007). The WAB-AQ was incorporated as a categorical variable to diverge from the assumption of a linear relationship between severity and treated recovery in favor of allowing flexibility in recovery across levels of severity. Prior to entry into the GLMs, each of the continuous variables was centered by subtracting its mean $\bar{x}$ from each individual observation.

Model accuracy was estimated using a leave-one-out cross-validation (LOOCV). One participant was set aside, and the model was trained on behavioral data from *N* – 1 participant. Then, the trained model was used to predict change in naming for the left-out participant. This procedure was repeated *N* times to yield a unique prediction for each participant. Performance of the LOOCV models was assessed by computing *R* ^2^ for the size of the residuals from the model compared to the size of the residuals from a null model where all predicted values are equal to the mean value of $\bar{y}$ (Alexander et al., 2015).

All statistical analyses were carried out using R software (Version 3.6.0). GLMs were constructed using the *glm*, *caret*, and *MASS* packages.

## Results

**Figure 3.**
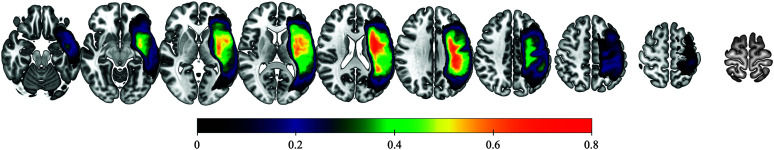
Lesion overlap map. Color bar represents proportional overlap (0%–80%). The lesion overlay map follows radiological convention.

Table 1 presents primary characteristics and demographic information for the 102 individuals who completed all therapy procedures and at least one outcome assessment. Figure 3 illustrates lesion distribution of the study sample.

**Table 1.** Baseline demographics and clinical characteristics.

Variable	Mean/count	*SD*	Range
Age (years)	60.5	10.9	29–80
Female, *n* (%)	42 (41.2)		
Non-Hispanic White, *n* (%)	75 (73.5)		
Education (years)	15.4	2.3	12–20
Time since stroke onset (months)	46.2	48.1	12–241
NIH Stroke Scale score	6.2	3.7	0–16
WAB-R Aphasia Quotient	60.0	22.7	14.5–93.1
Baseline PNT correct	79.9	60.9	0–172
WAIS Matrix Reasoning	12.0	5.7	3–22
ASRS severity	1.6	1.6	0–4
PPTT total	45.3	5.6	14–52
PRT correct	110.0	55.6	0–175
Right-handed, *n* (%)	90 (88.2)		
History, *n* (%)			
Diabetes	23 (22.5)		
Depression	24 (23.5)		
Aphasia type, *n* (%)			
Anomia	29 (28.4)		
Broca's	45 (44.1)		
Conduction	16 (15.7)		
Global	4 (3.9)		
Transcortical motor	1 (1.0)		
Wernicke's	7 (6.9)		

*Note.* NIH = National Institutes of Health (max score = 42); WAB-R = Western Aphasia Battery–Revised (max score = 100; cutoff = 93.8); PNT = Philadelphia Naming Test (max score = 175); WAIS = Wechsler Adult Intelligence Scale (max score = 26); ASRS = Apraxia of Speech Rating Scale (max score = 4); PPTT = Pyramids and Palm Trees Test (max score = 52); PRT = Philadelphia Repetition Test (max score = 52).

### Aim 1: Immediate Effects and Maintenance of Therapy Effects

**Figure 4.**
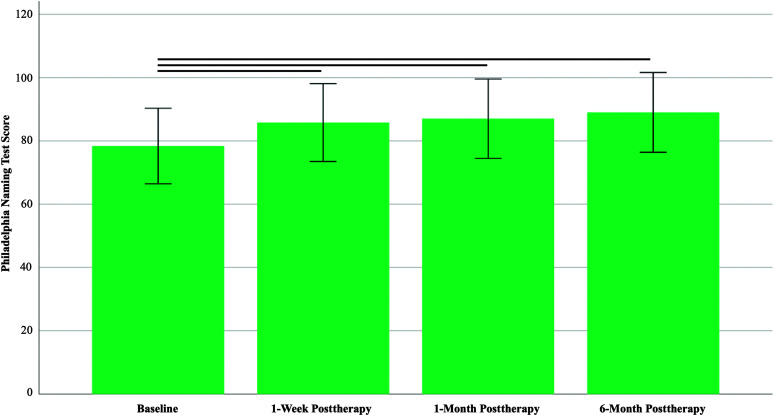
Change in primary outcome measure across time. Whiskers denote standard errors.

Participants correctly named 79.9 ± 60.9 items at baseline on average. One week after therapy, there was a significant increase in untrained items named correctly on the PNT (mean difference: 5.9 ± 10.4, Cohen's *d* = 0.56; paired *t*(101) = 5.69, *p* < .001). Therapy gains were maintained at follow-up assessments 1 month (mean difference: 6.4 ± 9.8, *d* = 0.66; paired *t*(99) = 6.57, *p* < .001) and 6 months (mean difference: 7.5 ± 11.5, *d* = 0.65; paired *t*(95) = 6.41, *p* < .001) after therapy compared to baseline performance. No significant differences in naming performance were observed between outcome assessments 1 week, 1 month, and 6 months and 6-month posttherapy (all *p*s > .100). Figure 4 shows naming performance across time points.

### Aim 2: Factors Associated With Therapy Response

**Figure 5.**
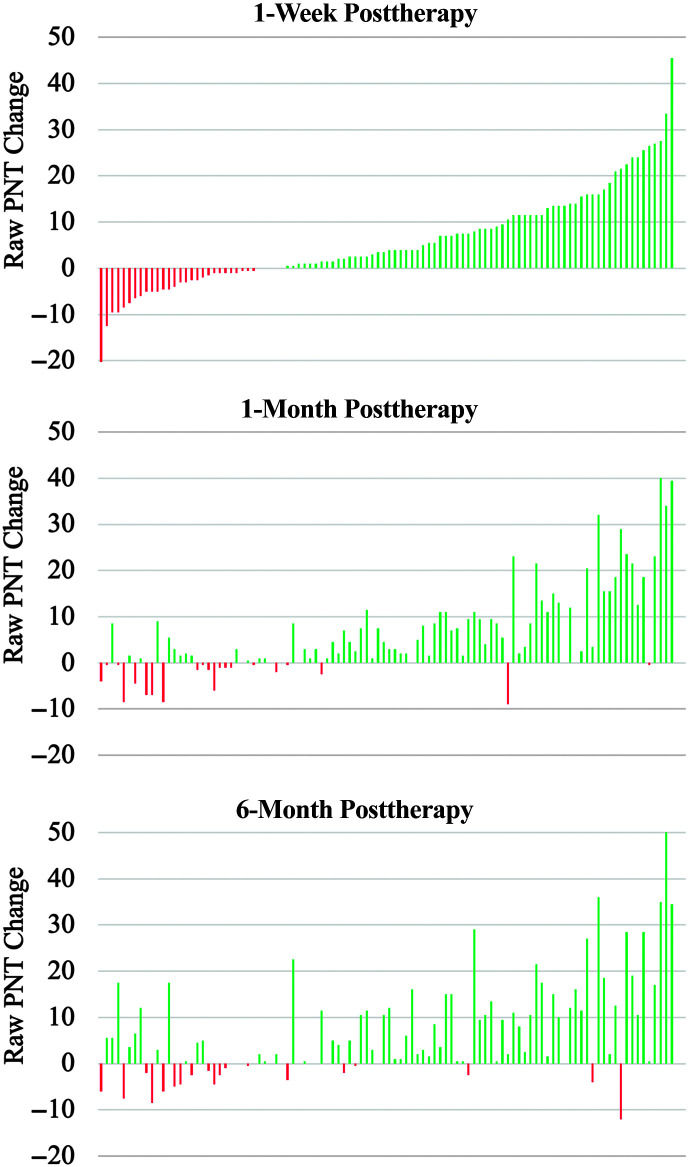
Distribution of change in naming performance across participants. Raw change scores are calculated with reference to baseline and ranked from lowest to highest immediately after therapy. PNT = Philadelphia Naming Test.

There was considerable heterogeneity in therapy response at the individual level. In terms of absolute change in performance from baseline, 69/102 individuals (67.6%) named more items correctly 1 week posttherapy compared to baseline. At the 1- and 6-month follow-up assessments, the corresponding numbers were 74/100 (74.0%) and 71/96 (74.0%), respectively. The distribution of individual change scores is presented in Figure 5.

In order to inform individual differences in therapy response, we examined the relationship between five factors that have been suggested to be associated with therapy response in prior research and therapy outcome at each time point. Both the WAB-AQ (ρ = .91, *p* < .001) and the WAIS Matrix Reasoning score (ρ = .31, *p* = .002) were positively correlated with the baseline PNT score. The factors were largely unrelated to one another, aside from the WAIS Matrix Reasoning score, which correlated positively with the WAB-AQ (*r* = .37, *p* < .001) and education (*r* = .34, *p* < .001) and negatively with age (*r* = −.27, *p* = .006).

*1 week posttherapy*. WAB-AQ severity (*p* < .001) emerged as the only factor independently associated with therapy response 1 week after treatment while adjusting for variability accounted for by the other four factors. The effect of each level of severity varied considerably. *Very severe* (ß = 0.20, *t* = 0.06, *p* = .957) and *severe* (ß = 2.51, *t* = 1.25, *p* = .215) aphasia were not associated with therapy gains, whereas *moderate* (ß = 9.65, *t* = 5.58, *p* < .001) and *mild* (ß = 5.85, *t* = 3.14, *p* = .002) aphasia were positively associated with change in naming at 1 week posttherapy. No other factors were statistically significant in the model (all *p*s > .10; model *R* ^2^ = .11).

*1 month posttherapy*. At 1 month after therapy, WAB-AQ severity (*p* < .001) was a significant predictor of change in naming while adjusting for variability accounted for by other predictors. A similar pattern was observed; *very severe* (ß = 0.32, *t* = 0.10, *p* = .921) and *severe* (ß = 1.31, *t* = 0.73, *p* = .470) aphasia were not associated with therapy outcomes, whereas *moderate* (ß = 11.54, *t* = 7.49, *p* < .001) and *mild* (ß = 6.47, *t* = 3.96, *p* < .001) aphasia were positively predictive of outcomes. Age (ß = −0.16) emerged as an influential inverse predictor but marginally failed to reach statistical significance at *p* < .05 (*t* = −1.88, *p* = .064). TPO, education, and cognitive reserve (WAIS) were not statistically significant in the model (all *p*s > .50; model *R* ^2^ = .21).

**Figure 6.**
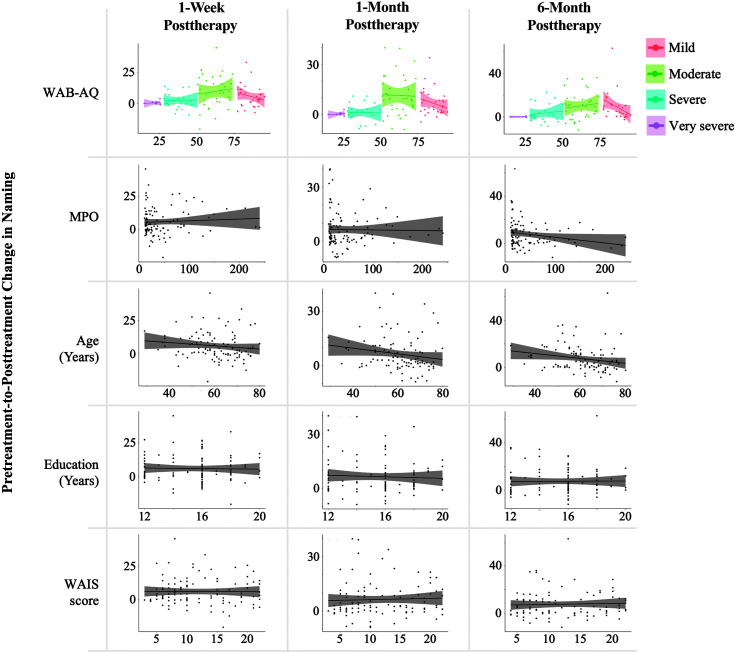
Independent association between prognostic factors and raw change in naming performance. WAB-AQ = Western Aphasia Battery–Aphasia Quotient; MPO = months postonset; WAIS = Wechsler Adult Intelligence Scale.

*6 months posttherapy*. Two of the five factors were independently associated with naming gains in the presence of the other factors 6 months after therapy, and a marginal effect of the three other factors was observed: WAB-AQ severity (*p* < .001), TPO (*p* = .033), and age (*p* = .059). *Moderate* (ß = 10.30, *t* = 5.52, *p* < .001) and *mild* (ß = 9.02, *t* = 4.33, *p* < .001) aphasia were predictive of a positive response to therapy, but therapy response was not influenced by *very severe* (ß = 0.21, *t* = 0.05, *p* = .963) and *severe* (ß = 3.37, *t* = 1.49, *p* = .139) aphasia. Both TPO (ß = −0.05, *t* = −2.16) and age (ß = −0.20, *t* = −1.91) were inversely predictive of naming outcomes. Education and cognitive reserve (WAIS) did not contribute significantly to the model (both *p*s > .50; model *R* ^2^ = .16). All models are shown in Table 2. Figure 6 represents the independent relationship between each prognostic factor and raw change in confrontation naming.

**Table 2.** General linear models for change in naming.

Factor	Estimate	*SE*	*t*	*p*
1 week posttherapy (*R* ^2^ = .110; total/residual *df* = 102/94)				
WAB-AQ severity^a^				< .001**
Very severe	0.20	3.64	0.06	.957
Severe	2.51	2.01	1.25	.215
Moderate	9.65	1.73	5.58	< .001**
Mild	5.85	1.86	3.14	.002**
TPO	0.01	0.02	0.50	.620
Age	−0.14	0.10	−1.45	.151
Education	0.01	0.47	0.02	.987
WAIS	−0.14	0.21	−0.65	.515
1 month posttherapy (*R* ^2^ = .214; total/residual *df* = 100/92)				
WAB-AQ severity^a^				< .001**
Very severe	0.32	3.20	0.10	.921
Severe	1.31	1.80	0.73	.470
Moderate	11.54	1.54	7.49	< .001**
Mild	6.47	1.63	3.96	< .001**
TPO	−0.01	0.02	−0.38	.707
Age	−0.16	0.09	−1.88	.064^†^
Education	−0.10	0.42	−0.24	.815
WAIS	−0.05	0.19	−0.25	.800
6 months posttherapy (*R* ^2^ = .156; total/residual *df* = 96/88)				
WAB-AQ severity^a^				< .001**
Very severe	0.21	4.55	0.05	.963
Severe	3.37	2.25	1.49	.139
Moderate	10.30	1.87	5.52	< .0001**
Mild	9.02	2.08	4.33	< .001**
TPO	−0.05	0.02	−2.16	.033*
Age	−0.20	0.11	−1.91	.059^†^
Education	0.26	0.52	0.51	.614
WAIS	−0.11	0.24	−0.48	.634

*Note.* WAB-AQ = Western Aphasia Battery–Aphasia Quotient; TPO = time postonset; WAIS = Wechsler Adult Intelligence Scale.

a Fixed effects significance derived based on chi-square test.

* *p* < .05.

** *p* < .01.

† *p* < .10.

**Figure 7.**
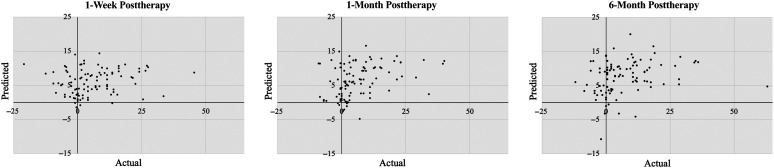
Scatter plot of actual versus predicted Philadelphia Naming Test scores based on leave-one-out cross-validation models.

Model performance for each time point was assessed using LOOCV. Prediction accuracy 1 week after therapy was estimated by computing *R* ^2^ for variability in naming outcomes accounted for by the LOOCV model (as per Alexander et al., 2015). Following this rigorous approach, the model did not account for any variability in change scores at 1 week posttherapy (*R* ^2^ = .00; Pearson's *r* for actual vs. predicted scores = .17; root-mean-square error [RMSE] = 10.4; mean absolute error [MAE] = 7.6). At the 1-month follow-up, the model accounted for 12% of variability in naming outcomes (*r*_actual/predicted_ = .36; RMSE = 9.1; MAE = 6.5). At the 6-month follow-up, the model accounted for 7% of variability in naming outcomes (*r*_actual/predicted_ = .29; RMSE = 11.2; MAE = 8.1; see Figure 7).

## Discussion

The primary purpose of the present study was to demonstrate immediate and long-term response to evidence-based anomia treatment and investigate the association of several potential prognostic factors and aphasia therapy outcome. To address this aim, we used data from the recently completed POLAR trial. Major components of the POLAR protocol were described in detail, including its purpose, recruitment, assessments, and therapy tasks. Briefly, we found that participants showed substantial average improvement in naming performance immediately after therapy and at follow-up assessments 1 month and 6 months after therapy. Nonetheless, considerable variability in therapy response was observed at the individual level. Aphasia severity was found to be strongly associated with naming outcomes at all time points. Participants with mild and moderate aphasia showed greatest naming recovery, whereas those with severe or very severe aphasia showed little or no response to therapy. Age emerged as a prognostic factor for outcomes at 1 month and at 6 months, and TPO similarly improved prediction accuracy for naming improvement 6 months after therapy. The implications of these findings are discussed in detail below.

### Change in Language Performance

The POLAR trial was designed to study predictors of language recovery by applying evidence-based aphasia therapy but not to address questions on treatment efficacy. Nonetheless, we analyzed the change in naming performance from pre- to posttherapy to glean the *assumed* effects of treatment at the group level. Considering the study design, this analysis should be regarded as ancillary, and the results ought to be interpreted with caution.

We observed a significant improvement in naming performance 1 week after therapy compared to baseline performance (*p* < .001), consistent with numerous studies suggesting that impairment-based SLT is an effective approach to improve language function in chronic aphasia (Brady et al., 2012, 2016; Breitenstein et al., 2017; Fridriksson et al., 2012; Leff et al., 2021; Wisenburn & Mahoney, 2009). Critically, a similar pattern of improvement was reported 1 month and 6 months after completion of therapy (both *p*s < .001). There was not a significant difference between the posttherapy assessments, suggesting robust maintenance of therapy gains. Several prior studies have examined long-term maintenance of therapeutic effects, but mixed results have been reported (Breitenstein et al., 2017; Efstratiadou et al., 2018; Kendall et al., 2019; Kendall & Nadeau, 2016; Menahemi-Falkov et al., 2022; Nickels, 2002; Wisenburn & Mahoney, 2009). In the largest aphasia therapy trial conducted to date, Breitenstein et al. (2017) found that therapy effects were maintained up to 6 months after 3 weeks of intensive SLT in 158 individuals. Similarly, Kendall et al. (2019) reported maintenance of language gains 3 months after completion of anomia therapy in 58 individuals. On the contrary, in a review of studies that have applied SFA therapy, Efstratiadou et al. (2018) observed maintenance effects only in half of participants in follow-up assessments 2–4.5 months after therapy. Most recently, Menahemi-Falkov et al. (2022) have conducted a systematic review of maintenance following intensive therapy programs in chronic aphasia and concluded that only 22% of individuals demonstrated long-term maintenance effects after aphasia therapy.

In light of these mixed findings, our findings of considerable improvement in untreated naming maintained 6 months after therapy completion in a comparatively large study sample are promising. Presently, most individuals with aphasia receive the majority of their therapy in the acute and subacute phases of recovery (Katz et al., 2000). This reality persists despite failure of recent randomized controlled trials to show benefit of SLT initiated during these phases (e.g., Godecke et al., 2021; Laska et al., 2011; Nouwens et al., 2017). The reason for this may rest on the notion that language recovery plateaus after the first few months from stroke onset, a myth that has effectively been debunked (e.g., Holland et al., 2017; L. Johnson et al., 2019). In fact, L. Johnson et al. (2019) showed that time spent in SLT was the strongest predictor of positive changes in language function even years after stroke, with no apparent ceiling effects. Therefore, our findings may provide promising evidence for the beneficial effects of restorative language therapy without the constraint of a specific time frame and, consequently, deliver hope to millions of individuals living with chronic aphasia today.

Notwithstanding, consistent with prior research (e.g., Code et al., 2010), therapy response was tremendously variable at the individual level. Immediately after therapy, 32.4% of participants failed to name more items than at baseline, whereas 26.0% of participants failed to name more items at 1 month and 6 months posttherapy. These proportions correspond to response patterns observed in prior studies (e.g., Breitenstein et al., 2017; Fridriksson, 2010; J. P. Johnson et al., 2020). It is unclear why some individuals do not seem to respond to conventional SLT. A post hoc analysis revealed that nonresponders at the final outcome assessment presented with more severe aphasia (WAB-AQ: 47.2 vs. 65.3, *p* < .001) were older (65.8 years vs. 58.7 years, *p* = .005) and had lower baseline PNT scores (50.9 vs. 92.2, *p* < .001). These results echo the findings reported under the first aim with respect to potential prognostic factors.

Various other variables not systematically manipulated here may similarly influence therapy response for some individuals. These include, but are not limited to, therapy dose and intensity, therapy modality (e.g., some individuals might benefit from the addition of tablet-based therapy or group therapy), and therapy focus. For instance, recent work from our lab suggests that while some individuals respond similarly to semantically and phonologically focused SLT, others may respond selectively to one therapy type and not the other (Kristinsson, Basilakos, et al., 2021). Furthermore, personality traits such as resilience, motivation, and social support cannot be overlooked. In addition, these variables may interact with each other to support or deter the potential for recovery in individual cases. The multiple dimensions that influence treated recovery highlight the need to move toward personalized medicine in aphasia therapy.

### Prognostic Factors

Our second aim investigated the association between aphasia severity, age, TPO, education, cognitive reserve, and naming outcomes. We focused our analyses on these particular factors as they have been subject to similar examinations in prior research, without firm conclusions as to how they can be used to inform clinical prognostication. Our findings revealed that aphasia severity, as measured by the WAB-AQ, emerged as a dominating predictor in each model. Age was found to be a negative predictor of change in naming from baseline to follow-up assessments at 1 month and 6 months posttherapy. TPO was only found to be predictive of change in naming performance at 6 months posttherapy. Cognitive reserve, as measured by the Matrix Reasoning subtest of the WAIS, and years of education were not associated with a treatment-induced change in naming at any assessment time point.

*Aphasia severity*. Aphasia severity is commonly recognized as an influential factor for language recovery (e.g., Kristinsson et al., 2022; Plowman et al., 2012; Watila & Balarabe, 2015). The cortical organization of language relies on dynamic interactions across temporal, parietal, and frontal brain regions (Hickok & Poeppel, 2007; Tourville & Guenther, 2011). As such, aphasia is best described as a network disorder at the cortical level (Fridriksson et al., 2018; Mesulam, 1990). Correspondingly, the severity of language deficits depends not only on both lesion size and location but also on the functional and structural integrity of the remaining intact brain tissue (Carter et al., 2012; Hope et al., 2016; Kristinsson, Zhang, et al., 2021; Kuceyeski et al., 2016). Consequently, reorganization of language relies on the extent of intact brain tissue (Kiran & Thompson, 2019), particularly in residual language regions and regions immediately surrounding the necrotic lesion (Fridriksson et al., 2012).

The nature of the relationship between severity and treated recovery has, nonetheless, been debated (e.g., Persad et al., 2013). Some researchers have found a relative advantage for individuals with milder aphasia (e.g., Code et al., 2010; Efstratiadou et al., 2018), but an advantage for individuals with more severe aphasia has also been reported (e.g., Robey, 1998). Our results fall closely in line with the former view. One week after therapy, participants with very severe (i.e., WAB-AQ = 0–25) and severe (WAB-AQ = 26–50) aphasia experienced insignificant improvement by 0.20 and 2.51 PNT items (both *p*s > .20), respectively, while adjusting for the effects of other model terms (TPO, age, education, and WAIS/cognitive reserve). On the other hand, participants with mild (WAB-AQ ≥ 76) and moderate (WAB-AQ = 51–75) aphasia showed a robust improvement of 9.65 and 5.85 PNT items, respectively (both *p*s < .01). A similar pattern of nonlinear effect of aphasia severity on treated recovery was observed at both 1 month and 6 months after therapy. Therefore, our results strongly suggest that better language performance at baseline is associated with favorable response to naming therapy, whereas individuals with very severe aphasia may not benefit from conventional impairment-based SLT of the type that was employed in this study. It is important to caution that these findings do not support the conclusion that severe aphasia is untreatable, but rather that naming therapy administered for 30 hr does not improve lexical–semantic access as measured by the PNT. Severe aphasia might require training for a longer period of time, with a different dosage or a different outcome measure to show benefit. Thus, future research should aim to characterize in greater detail the relationship between severity and therapy response, with the intention of generating benchmarks that can be used to guide clinical decision making. Furthermore, the nature of the relationship between severity and therapy outcomes observed here suggests that future research should not assume a positive linear effect of severity but rather allow greater flexibility when characterizing this association.

*TPO*. TPO emerged as a significant predictor only of change in naming from baseline through the 6-month follow-up assessment. Adjusting for variability accounted for by the other four factors, each additional month postonset was associated with .05 item less improvement in naming. These findings are largely consistent with prior research suggesting minimal effect of time beyond the first year of recovery (Holland et al., 2017; Moss & Nicholas, 2006; Nardo et al., 2017; Persad et al., 2013). Importantly, the study sample included participants with a wide range of TPO, including nine participants who were at least 10 years out from their last stroke. Reassuringly, these participants showed a similar immediate treatment response to the rest of the sample (PNT change: ≥ 10 years vs. < 10 years, 7.8 vs. 5.7, *p* < .553), supporting the notion that there is no inherent point of plateau for long-term language recovery (Holland et al., 2017; L. Johnson et al., 2019). However, the former group showed a trend toward a decrease in change scores across time (1 month: 7.33; 6 months: 4.61), whereas the opposite trend was detected in participants with shorter TPOs (1 month: 6.32; 6 months: 7.83). Thus, while TPO does not appear to be a prognostic factor for immediate treatment response, future research must determine whether time from stroke is an influential factor for long-term maintenance of therapeutic effects.

*Age*. Age was a consistent negative predictor of performance 1 month and 6 months after therapy. Specifically, each additional year was associated with decreased change in naming by .16 and .20 items at 1 month and 6 months after therapy, respectively. Although age was not a significant predictor in the model for change in naming 1 week posttherapy, the direction of the effect was the same as at 1 and 6 month posttherapy (ß = −0.14, *p* = .151; see Table 2). Normal aging is generally accompanied by a gradual reduction in neural plasticity (Toth et al., 2008; Vara et al., 2003), which intuitively could lead to a decreased capacity for language recovery. Several prior studies have yielded findings consistent with the notion that younger individuals show greater language recovery than older individuals (Lendrem et al., 1988; R. C. Marshall et al., 1982; Nakagawa et al., 2019; Pickersgill & Lincoln, 1983; van de Sandt-Koenderman et al., 2008). However, multiple studies have also failed to find a consistent relationship between age and treated recovery (Code et al., 2010; Nardo et al., 2017; Persad et al., 2013; Seniów et al., 2009). Due to these mixed findings, the association between age and recovery has been described as equivocal in recent systematic reviews (Ellis & Urban, 2016; Watila & Balarabe, 2015). Our findings provide support for the former view, in a sample considerably larger than recruited in the studies cited above; age has a subtle but meaningful effect on treated language recovery.

*Cognitive reserve*. WAIS Matrix Reasoning score, as a measure of nonverbal reasoning skills, was not found to be associated with the degree of treated recovery. This finding contrasts the results reported in several recent studies (e.g., Dignam et al., 2017; Fillingham et al., 2006; Lambon Ralph et al., 2010; Seniów et al., 2009). For instance, Dignam et al. (2017) found that verbal short-term memory predicted both naming performance after treatment and maintenance of language gains following anomia therapy that, in principle, was comparable to the therapy applied here. There are several potential reasons for the discrepancy in findings. First, Dignam et al. implemented a different measure of cognitive ability. In particular, the measure found to be predictive of treatment outcomes relied on a verbal task, whereas the WAIS Matrix Reasoning task used in this study measures nonverbal cognitive function. It is inherently challenging to measure cognitive abilities in persons with aphasia due to the language barrier, and reliance on verbal tasks may favorably bias participants with milder language impairment. Second, participants in Dignam et al.'s study received more hours of treatment (48 vs. 30), which may inevitably influence language recovery. Relatedly, therapy success was evaluated on treated items, as opposed to untreated items here. It is possible that higher order cognitive factors support implicit learning specific to trained items. Last, Dignam et al.'s study included substantially fewer participants (*N* = 34), which is a common issue in the aphasia therapy literature that can inflate both negative and positive research findings (e.g., Lorca-Puls et al., 2018).

Cognitive reserve, like residual language function, depends largely on the integrity of remaining intact brain tissue (e.g., Fonseca et al., 2019). Therefore, it would not be unreasonable to expect a relationship between cognitive reserve and therapy outcomes. Our results showed that the WAIS score was positively correlated with the baseline PNT score (*r* = .31, *p* = .002) and positively, albeit nonsignificantly, correlated with the raw change in naming from baseline to each outcome time point (all *p*s > .20; see Figure 6). Furthermore, we observed a positive correlation between WAIS score and aphasia severity (WAB-AQ; *p* < .001) and a negative correlation with age (*p* = .006). Thus, any potential effect of cognitive reserve on naming outcomes may have been fully accounted for by aphasia severity and age. Future research will need to map out in greater detail the relationship between different aspects of cognition and aphasia severity to inform the association between cognitive reserve and treatment outcomes.

*Education*. Finally, we found no association between education and treated recovery. Education attainment has been associated with cognitive reserve in older individuals (Foubert-Samier et al., 2012; Staff et al., 2004), which has led some researchers to postulate whether more formal education serves to support better language recovery in stroke (Connor et al., 2001; González-Fernández et al., 2011; Perneczky et al., 2007). Several studies have reported a relationship between education and degree of language recovery (Connor et al., 2001; González-Fernández et al., 2011; Hillis & Tippett, 2014; Ramsey et al., 2017; Smith, 1971), whereas others have failed to find a consistent association (e.g., Helm-Estabrooks, 2002; Hillis et al., 2018; Lazar et al., 2008). Even if education was positively correlated with cognitive reserve in this study (*p* < .001), our results do not provide any indication in support of the view that this association supports treated language recovery.

In summary, we observed immediate improvement in naming performance after SLT and retention of language gains 6 months after treatment in a representative sample of persons with aphasia. Our results indicate that individuals with relatively mild language impairment (i.e., WAB-AQ ≥ 51) are more likely to respond favorably to impairment-based naming therapy compared to those with more severe language deficits. Younger individuals are more likely to retain therapy-induced language gains in the long term compared to older individuals, and shorter TPO may similarly confer positive long-term prognosis. Of equal importance, we found that the very severely affected group (i.e., WAB-AQ ≤ 25) and, to a lesser extent, the severely affected group (i.e., WAB-AQ = 26–50) did not demonstrate a significant improvement in naming performance after therapy, suggesting that an alternative form of treatment might need to be considered for this group. These findings are consistent with the recent notion that greater focus should be placed on counseling and alternative and augmentative and alternative approaches in severe aphasia, whereas restoring language function should be a primary aim in moderate and mild aphasia (Fridriksson & Hillis, 2021).

This study represents the largest systematic examination of the direct association between five commonly considered prognostic factors and response to therapy. Our results certainly suggest that it is possible to enhance clinical prognostication in aphasia, and future research should aim to determine in greater detail the extent to which therapy response can be informed based on baseline aphasia severity, age, and TPO. Looking further ahead, a prognostic model based on these factors may (a) serve as a basis to test the unique predictive value of other prognostic factors (e.g., lesion information or language deficit profiles) and (b) be utilized to examine differences related to therapy parameters such as treatment concentration or focus, intensity or dosage, and therapy modality.

### Limitations

There are several important limitations that require discussion. First, we focused on naming as the primary outcome measure. Some authors have criticized the use of naming outcomes in aphasia therapy research based on the view that the ability to name objects does not reflect the multifaceted nature of human communication (e.g., Webster et al., 2015). While we concur with this view in part, we favor the use of a naming measure because anomia (i.e., word-finding difficulties) is present in all types of aphasia (Goodglass & Wingfield, 1997) and is a common target in aphasia therapy (e.g., all therapy tasks applied here), naming performance correlates strongly with aphasia severity (here, *r* = .90, *p* < .001) and discourse measures (mean content words per minute, propositional density, and verbs per utterance derived from picture description [broken window], Cinderella story retelling, and PB&J [peanut butter and jelly sandwich] procedural description; *r* = .35–.52, all *p*s < .001; see Kristinsson, Basilakos, et al., 2021), and anomia therapy progress has been shown to induce multiple levels of generalization (e.g., Gilmore et al., 2020; Madden et al., 2021).

Second, although we aimed to recruit a representative sample of persons with chronic aphasia, those most severely affected were excluded as they were unlikely to be able to participate fully in the therapy tasks. Along a similar vein, it is possible that the participants who entered the study were relatively motivated (i.e., searching for resources) and/or had a social network in place that supported them in pursuing therapy. Both aspects may have introduced an inherent sampling bias. In addition, the number of items trained in each therapy session varied depending on performance, which unavoidably may correlate with severity of aphasia symptoms. Notwithstanding, despite these challenges, our sample represented the heterogeneous nature of this population as well as reasonably possible.

Third, as the POLAR trial was not designed to examine the efficacy of aphasia therapy, we cannot definitively conclude that the average improvement in naming performance pre- to posttherapy was a result of the therapy tasks administered here. While it is highly unlikely to observe the positive changes reported under Aim 1 by chance, it is certainly possible that factors other than the therapy tasks may have influenced these changes. For instance, engaging with the clinicians and other study participants outside of the treatment room may have forced participants to hone their functional communication skills more than they would have outside of the trial. Nonetheless, the therapy tasks were selected based on efficacy established in prior research, and these particular tasks were intended to induce language gains robust enough to enable examination of prognostic factors. Thus, we explicitly assume that the language gains observed here are the result of the therapy participants underwent.

Last, our examination of prognostic factors is by no means exhaustive. The factors investigated here were chosen as they have frequently been implicated as potentially important for understanding therapy response in aphasia, but prior literature is ripe with mixed and, sometimes, contrasting findings. In an effort to elucidate the relationships between these factors and treated recovery, we therefore asked questions that have been asked before, but in a much larger sample. Future research should focus on other sources of variability, such as lesion location, genetic factors, specific language domains, and the interactions between these variables. As the field moves toward personalized medicine, it will be critical to disseminate how multiple different variables independently and in tandem may inform clinical decision making with the clients' interests at the forefront.

## Conclusions

This study described the protocol of POLAR, a recently completed, large-scale therapy trial in aphasia, and examined the immediate and longitudinal change in naming performance after treatment, as well as the predictive value of several frequently studied prognostic factors. In conclusion, we observed a substantial improvement in naming performance after 30 hr of impairment-based therapy compared to before therapy and throughout follow-up assessments 1 month and 6 months after therapy completion. The extent of language recovery was influenced by initial aphasia severity, age, and time poststroke. These results are highly encouraging as the prevalence of chronic aphasia continues to rise. The predictability of therapy response is a fundamental prerequisite for the development of personalized therapy planning in service provision in aphasia. Future studies should capitalize on these findings to substantiate a prognostication framework that can be subjected to empirical testing.

## Data Availability Statement

In accordance with the National Institutes of Health policy for data sharing (http://grants.nih.gov/grants/policy/data_sharing/index.htm), upon completion of the POLAR (Predicting Outcomes of Language Rehabilitation) trial and dissemination of primary study results, the analysis data files will be made available to the public, along with the final version of the study protocol, the data dictionary, and brief instructions. The data that support the findings of this study are available from the corresponding author upon reasonable request.

## Supplementary Material

10.1044/2022_JSLHR-22-00347SMS1 Supplemental Material S1 Participants' cognitive-linguistic test scores.
