# Supplementary material for: Predicting Outcomes of Language Rehabilitation: Prognostic Factors for Immediate and Long-Term Outcomes After Aphasia Therapy
Source: J Speech Lang Hear Res. 2023 Feb 24;66(3):1068–84. doi: 10.1044/2022_JSLHR-22-00347 (PMC10205105; doi:10.1044/2022_JSLHR-22-00347)
Supplement: Supplemental Material S1 [file JSLHR-66-1068-s001.pdf]

**Assessment**	***n***	**Mean**	***SD***
WAB			
Aphasia Quotient	102	60.0	22.7
Spontaneous Speech	102	11.5	4.8
Auditory Comp.	102	7.7	1.8
Repetition	102	5.2	2.9
Naming	102	5.5	3.0
ASRS Apraxia of Speech score	101	1.6	1.6
WAIS	102	12.0	5.7
PPTT	102	45.3	5.6
KDT	102	45.9	5.6
PRT	101	110.0	55.6
Naming 40	85	16.5	13.3
NAVS subscores			
VNT	99	10.3	7.4
VCT	102	19.1	4.3
Argument Structure	97	16.9	12.8
ASPT	96	16.4	12.8
SPPT	96	9.6	10.7
SCT	102	21.8	5.8
TALSA			
W5SF	90	13.1	3.1
W5SU	90	14.9	3.4
NW5SF	89	13.6	2.9
NW5SU	89	14.3	3.3
Triplet 1	90	9.7	3.2
Triplet 2	90	9.3	3.2
PALPA			
PALPA 1	90	58.0	9.4
PALPA 2	90	59.9	9.5
PALPA 8	90	7.7	7.2
PALPA 14	90	9.8	4.0
PALPA 15	90	47.6	8.1
PALPA 16 ISW	87	18.3	6.9
PALPA 16 ISNW	87	8.0	3.6
PALPA 17 FSW	86	13.3	7.1
PALPA 17 FSNW	86	6.7	4.0
Discourse			
Mean CWPM	98	47.0	33.3
Mean PD	98	.4	.1
Mean VPU	98	.8	.5

Supplemental Material S1. Participants' cognitive-linguistic test scores. WAB = Western Aphasia Battery; ASRS = Apraxia of Speech Rating Scale; WAIS = Wechsler Adult Intelligence Scale; PPTT = Pyramids and Palm Trees Test; KDT = Kissing and Dancing Test; PRT = Philadelphia Repetition Test; NAVS = Northwestern Assessment of Verbs in Sentences; TALSA = Temple Assessment of Language and Short-term Memory in Aphasia; PALPA = Psycholinguistic Assessment of Language Processing in Aphasia.
